# The effect of duloxetine on postoperative pain and opium consumption in spine surgery: A systematic review

**DOI:** 10.1016/j.xnsj.2023.100303

**Published:** 2023-12-04

**Authors:** Reza Minaei Noshahr, Emad Kouhestani, Mohsen Dibamehr, Muntadhar Alshohaib

**Affiliations:** a Department of Orthopedic Surgery, Shahid Beheshti University of Medical Sciences, Velenjak Street, Tehran, Iran; b Bone Joint and Related Tissues Research Center, Akhtar Orthopedic Hospital, Shahid Beheshti University of Medical Sciences, Shariati street, Tehran, Iran

**Keywords:** Spine surgery, Duloxetine, Systematic review, Postoperative pain, Safety, Analgesic

## Abstract

**Background:**

Postoperative pain after spinal surgery is a major problem that can impact patients' quality of life. Duloxetine is a serotonin and norepinephrine reuptake inhibitor (SNRI) with analgesic effect in different pain disorders. In this study, we aim to evaluate the safety and analgesic effect of duloxetine on acute and chronic pain following spine surgery.

**Methods:**

A systematic search was completed on MEDLINE, PsycINFO, and Embase through OVID from inception to April 2023 to find relevant articles. We used Cochrane methodology to evaluate the bias of included studies. Investigated outcomes included postoperative pain, opioid consumption, and adverse events.

**Results:**

Seven articles involving 487 participants were included in our systematic review. Out of 7 papers, 5 were randomized clinical trials, 1 was a pilot trial and 1 was a retrospective observational study. The results of these studies indicated the analgesic effect of duloxetine on postoperative pain, which was measured using numeric rating scale, verbal numeric scale, brief pain inventory, and visual analogue scale. Duloxetine was generally safe without serious adverse events. The most common reported adverse events included headache, nausea, vomiting, itching, dizziness, and drowsiness.

**Conclusions:**

Duloxetine may be an effective treatment option for postoperative pain following spine surgery, but further rigorously designed and well-controlled randomized trials are required.

## Introduction

The development of minimally invasive procedures for spinal disorders has improved surgical outcomes, especially postoperative pain [1]. However, pain after spine surgeries remains a major concern [2]. Acute postoperative pain, a major challenge for orthopedic surgeons, is reported by 80% of patients that undergo spinal surgery of which 80% rate their pain as severe [3]. Postoperative pain can result in delayed recovery, increased risk of complications, and short and long-term morbidity [4]. Pain after spine surgeries can lead to chronic pain, which substantially affects the patient's quality of life [5]. Moreover, the nature of the pain following spine surgeries is neuropathic, hence, management of pain is more complex with standard analgesia [6]. Multimodal analgesia encompasses a comprehensive approach to pain management, involving a combination of medications, interventions, and therapies tailored to an individual's specific needs and circumstances. This strategy typically includes a range of pain-relief methods, such as opioids, nonopioid medications, regional anesthesia techniques, nerve blocks, and nonpharmacological interventions like physical therapy or acupuncture [7]. Ketamine and gabapentinoids, in particular, have been proven to lessen the requirement for opioids and to improve postoperative pain rating scales [8,9]. Opioids are a widely used effective method to control postoperative pain; however, adverse events such as respiratory depression, drowsiness, dizziness, nausea, and vomiting restrict their usage [6].

Antidepressants have been shown to reduce pain to varied degrees in a number of persistent and chronic pain disorders, including fibromyalgia, postherpetic neuralgia, and diabetic neuropathy [10,^9^,11]. Duloxetine is a potent selective serotonin and norepinephrine reuptake inhibitor that is primarily used in psychiatric disorders [12]. Several studies have indicated the analgesic effect of duloxetine in osteoarthritis, total knee arthroplasty, total hip arthroplasty, chronic low back pain, anterior cruciate ligament arthroscopy, and rotator cuff repair [13], [14], [15], [16], [17], [18], [19].

Duloxetine typically falls within a dose range of 40 to 60 mg per day for adults, usually taken once daily [20]. This drug undergoes extensive hepatic metabolism primarily mediated by enzymes like CYP1A2 and CYP2D6, yielding various metabolites, including the potent 4-hydroxyduloxetine [21]. With a half-life of approximately 12 hours, duloxetine provides sustained therapeutic effects, allowing for once-daily administration [12]. However, caution is crucial when considering its interactions with other medications, as it should not be used concomitantly with monoamine oxidase inhibitors or within 14 days of monoamine oxidase inhibitor discontinuation [22]. It may also interact with other serotonergic drugs, necessitating careful management to prevent serotonin syndrome [23]. Furthermore, potential interactions with drugs affecting liver enzymes should be taken into account, highlighting the importance of consulting a healthcare provider for personalized dosing and a comprehensive assessment of drug interactions based on individual medical profiles and concomitant medications.

There is limited evidence regarding the efficacy of duloxetine in the management of acute and chronic pain after spine surgeries. Current pain control strategies in spine surgery have several limitations due to their adverse events, therefore it is essential to evaluate new analgesic agents to improve the outcome of patients. Since the results of the available studies do not allow for the establishment of a firm conclusion, a systematic review is necessary to assess the effectiveness of duloxetine in spine surgery. In this paper, we aim to evaluate the efficacy of duloxetine on acute and chronic pain following spine surgeries. To the best of our knowledge, this is the first systematic review that specifically focuses on the efficacy of duloxetine in spine surgery.

## Method

### Literature search and selection criteria

A systematic search was conducted on 3 databases, MEDLINE, PsycINFO, and Embase, through OVID from inception to April 2023. We followed Preferred Reporting Items for Systematic Reviews and Meta-analyses guidelines for this systematic review [24]. Relevant studies were identified by searching the following keywords: duloxetine OR cymbalta AND spinal fusion or interbody fusion or laminectomy or lumbar surgery or spinal surgery or lumbar spine surgery or spine or spinal or spine surgery.

We also examined the reference lists of all included articles and Google Scholar to avoid missing relevant articles. There were no restrictions on publication date or status. We applied the following inclusion criteria for the search and study selection: (1) original studies that used duloxetine, (2) in patients that underwent spine surgery, (3) assessed chronic or acute pain as the primary outcome using different scales, and (4) full papers published in English. Systematic reviews, narrative reviews, meta-analyses, studies using the same dataset, letters to editor, commentaries, animal studies, case reports, case series, conference abstracts, and guidelines were excluded.

### Study selection

Two reviewers independently evaluated title/abstract and full-text of articles collected from the electronic search according to inclusion and exclusion criteria in order to identify potentially eligible papers. Discussion was used to settle disagreements between the 2 reviewers. If the full text was not available, it was requested from the authors online.

### Quality assessment

**Fig. 1:**
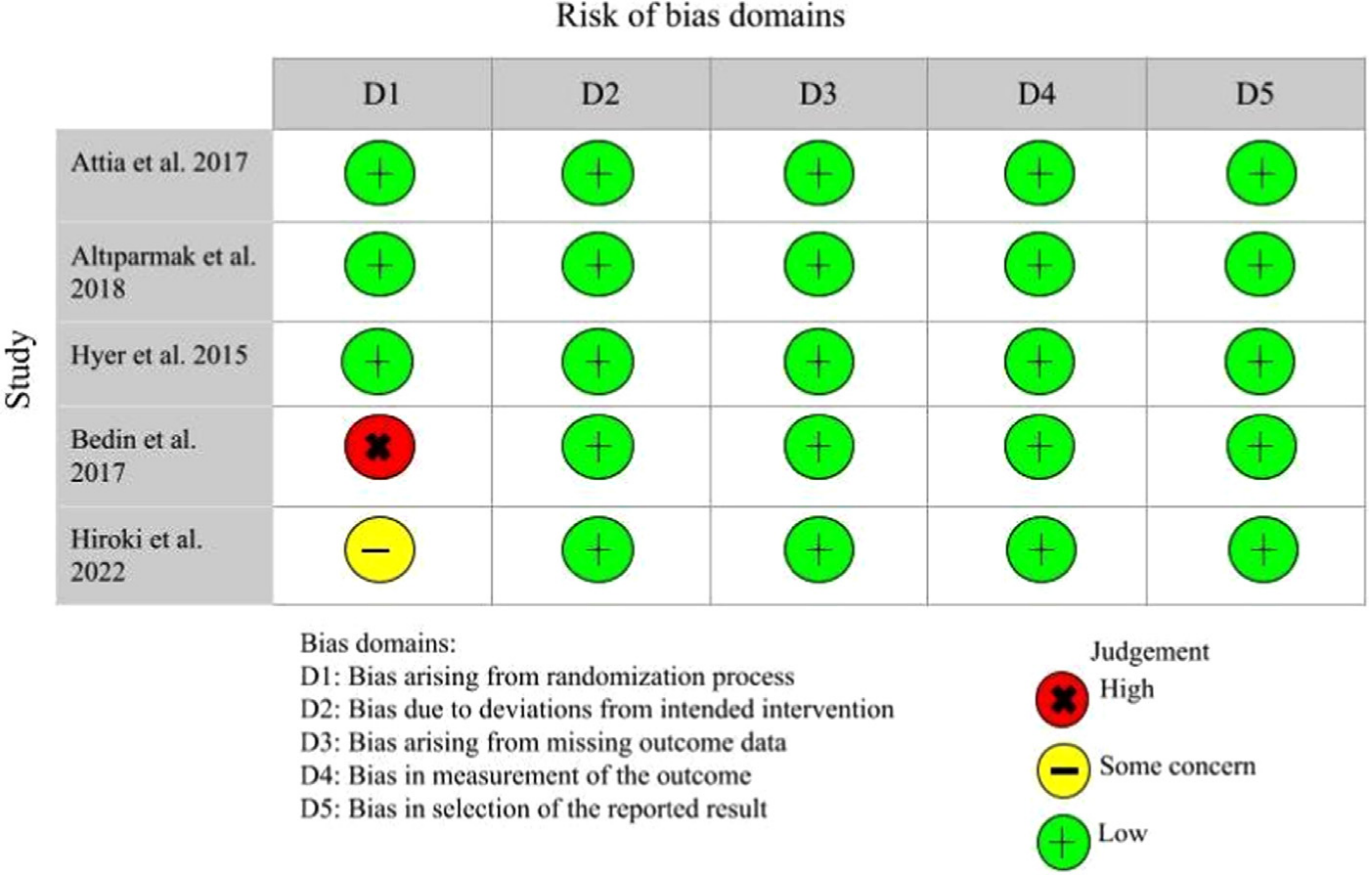
Methodological quality assessment of included studies.

Bias was examined using the Cochrane Collaboration's tool based on the following 5 domains: bias arising from the randomization process, bias due to deviations from intended interventions, bias due to missing outcome data, bias in measurement of the outcome, and bias in selection of the reported result. This was completed in accordance with the guidelines in the *Cochrane Handbook for Systematic Review of Interventions* [25]. To categorize high and low bias, if a study has adequately addressed these aspects and minimized potential biases, it is considered to have low bias. Conversely, if the study lacks proper procedures to mitigate bias, it falls into the high bias category (Fig. 1.).

### Data extraction

Two reviewers independently received the full-texts of eligible studies and extracted the following data from each article: name of first author, publication date, country, study design, intervention (dose, route of administration, duration of treatment, timeline for administering the medication), participants, characteristics of comparison/control group, results, protocol, outcome, surgery, and measure.

The data was obtained through 3 methods: extraction from the manuscript, conversion from provided tables, or reaching out to corresponding authors for their results if the previous approaches were unsuccessful. If requested, an Excel spreadsheet with the extracted data can be provided.

### Outcome definition

The primary outcome of our review was the effect of duloxetine on postoperative pain which was assessed by validated scales and collected as mean change from baseline to follow-ups. We also examined opioid consumption as an indicator of pain as well as adverse events across included studies.

## Results

### Search result

**Fig. 2:**
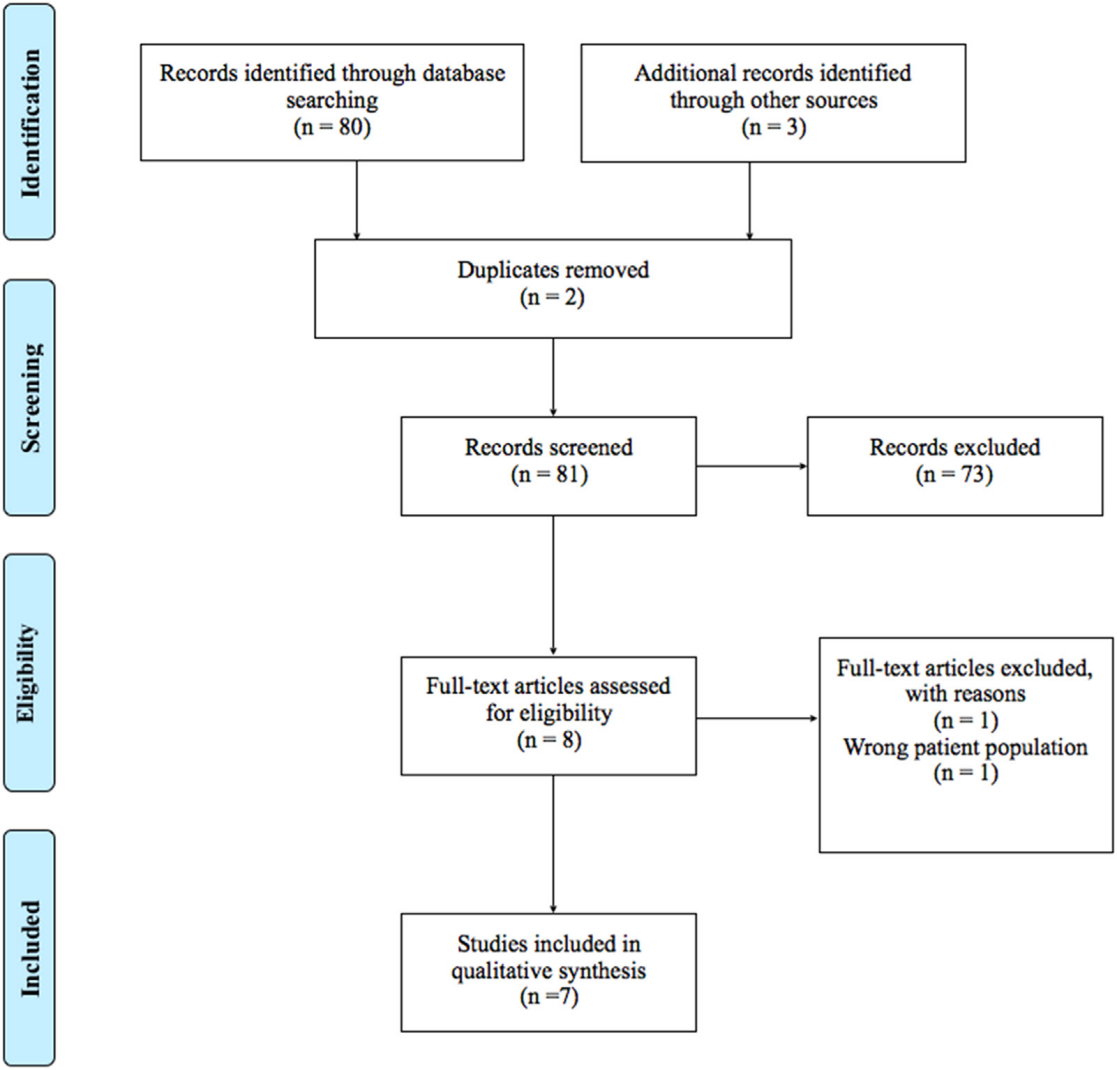
Flow diagram for the systematic review as per the Preferred Reporting Items for Systematic Reviews and Meta-Analyses (PRISMA).

The systematic search resulted in 80 records. We also found 3 records through manual search of relevant studies. After removing duplicates (n = 2), we excluded 73 records based on title/abstract screening. We reviewed the full texts of the remaining 8 papers in accordance with our inclusion criteria. After assessing the full texts, another 1 study was excluded; the population of the study did not meet the inclusion criteria for our review. We did not exclude any studies due to risk of bias. Finally, 7 articles were included in this systematic review [1,[26], [27], [28], [29], [30], [31]. Five randomized clinical trials (RCTs), 1 pilot trial and 1 retrospective observational study involving 487 participants were included in the current analysis (Fig. 2).

### Study characteristics

Five RCTs, 1 pilot trial, and 1 retrospective observational study involving 487 participants were included in the current analysis. Studies characteristics were extracted and are displayed in Table 1. All of the articles had been published since 2017, except 1 that had been published in 2015. The dosage of duloxetine ranged from 20 to 60 mg per dose across included studies. A single dose of 40 mg duloxetine 1 hour before surgery was administered in 1 study [31]. In 2 studies patients received 2 doses of duloxetine, with 2 studies using 1 dose of medication 1 hour before and 24 hours after surgery [27,28] and 1 study administered duloxetine 1 hour before and the morning after surgery [30]. Daily duloxetine for 3 months was administered in 2 studies [1,29]. In the 7 included studies, various types of spinal surgeries were investigated, including spinal cord stimulation [26], lumbar laminectomy [27], surgery for disc herniation [28], unspecified spine surgeries in 3 studies [1,^29^,30], and posterior lumbar interbody fusion surgery [31]. Numeric rating scale (NRS) [1,^26^,27], verbal numeric scale [28,29], brief pain inventory [30], visual analogue scale (VAS) [31], Pain catastrophizing scale [26] and McGill Pain Questionnaire (MPQ) [26] were used to determine postoperative pain in included studies. Three and 2 studies evaluated pain 48 hours [27,^28^,30] and 12 weeks [1,29] after surgery respectively.

**Table 1:** Study demographics.

Author	Country	Study design and participants	Intervention	Outcome	Measure	Results
Prabhala et al. [27]	USA	Pilot trial N = 108 (41 were on duloxetine and 67 were not) participants who underwent SCS	Duloxetine, oral, 30–60 mg	Pain 12 mo after surgery	NRS MPQ PCS	Patients receiving duloxetine and SCS (n = 41) had better pain relief in the affective component of MPQ than those receiving SCS alone (p = .046).
Attia et al. [28]	Egypt	RCT N = 120 participants who underwent Lumbar Laminectomy	4 groups: group P: placebo, group E: etoricoxib 120 mg, group D: duloxetine 60 mg, group D/E: duloxetine 60 mg + etoricoxib 120 mg. All drugs were given 1 h before surgery and 24 h after.	Pain 30 min after the end of anesthesia (time = 0), 2, 4, 6, 12, 24, and 48 h postoperatively Total morphine consumption (24 h and 48 h) The time to first rescue analgesic	NRS	Group D/E revealed a significant reduction in pain scores over the entire postoperative period at rest and on movement (p = .001).
Altıparmak et al. [29]	Turkey	RCT N = 6 participants who underwent spinal surgery due to disc herniation	3 groups: 1: pregabalin 75 mg 1 h before surgery, postoperative 12 h and 24 h. 2: duloxetine 60 mg 1 h before surgery, postoperative: 12 h, placebo, and, 3: at the 24 h, they received duloxetine 60 mg again.	Pain postoperative first min, 30 min, first h, and the 12h, 24h, and 48h.	VAS	The mean VAS scores of the pregabalin and duloxetine groups were similar to each other and less than control group (first min: D = 0 (0, 1), p = (0, 0), C = 0 (0, 1), p = .049, 30 min: D = 2 (2, 3), p = 2 (1, 2), C = 3 (2, 3), p = .001, 1 h: D = 3 (3, 4), p = 3 (3, 4), C = 4 (4, 5), p = .001, 24 h: D = 3 (2, 4), p = 3 (2, 4), C = 4 (4, 4), p = .001, 48 h: D = 2 (1, 3), p = 2 (1, 3), C = 3 (3, 4), p = .001).
Hyer et al. [30]	Georgia	RCT N = 68 participants who underwent spine surgery	1: Duloxetine + opioid, oral 2: placebo + opioid, treatment started 2 wk before surgery and continued for 3 mo	Pain prior to surgery, 4 wk postsurgery, and 12 wk postsurgery. Opioid consumption	BPI	Duloxetine reduced pain in the experimental group compared to the control group (p = .001).
Bedin et al. [31]	Brazil	RCT N = 57 participants who underwent spine surgery	1: placebo 2: 60 mg duloxetine 1 h before surgery and the following morning	Pain 2, 6, 12, 24, 36, and 48 h after surgery Total consumption of fentanyl 48 h after surgery	VNS	Significant difference in fentanyl consumption 24 and 48 h after surgery (p = .001). The pain scores over 48 h did not significantly differ between groups (p > .05).
Hiroki et al. [32]	Japan	RCT N = 46 participants who underwent posterior lumbar interbody fusion surgery	40 mg duloxetine or 4 mg diazepam (control) 1 h before surgery	Pain 2, 3, 5, 10, 18, 21, 27, 42, 45, and 51 h after surgery Fentanyl use 0–12, 12–24, 24–36, 36–48, and 0–48 h postoperatively Rate of analgesic adjuvant use	VAS	No significant difference between duloxetine and control groups (p = .139).
Tsuji et al.[1]	Japan	Retrospective observational N = 24 participants who underwent spinal surgery	Duloxetine 20–60 mg, more than 3 mo	Pain 3 mo post-treatment initiation Ongoing concomitant tramadol usage	NRS	Duloxetine was effective for all patients with postsurgical chronic pain.

SCS, spinal cord stimulation, NRS, numeric rating scale, MPQ, McGill Pain Questionnaire, RCT, randomized clinical trial, VAS, visual analogues scale, BPI, brief pain inventory, VNS, verbal numeric scale, PCS, pain catastrophizing scale.

### RCTs

Attia and Mansour [27] conducted an RCT and randomly assigned 120 patients to 4 groups (group P: placebo, group E: etoricoxib 120 mg, group D: duloxetine 60 mg, group D/E: duloxetine 60 mg + etoricoxib 120 mg). All drugs were given 1 hour before surgery and 24 hours after. Pain was assessed using NRS, 30 minutes after the end of anesthesia, 2, 4, 6, 12, 24, and 48 hours postoperatively. The results of study indicated that group D/E have significantly lower pain scores at rest than other groups across all time points (p = .001) [27]. Group E exhibited a significant reduction in pain scores at rest at all time points when compared to Group P, and at time points 0, 2, and 4 hours when compared to group D (p = .001). Additionally, Group D experienced a significant decrease in pain scores at 24 and 48 hours when compared to Group P (p = .001). During movement, pain was consistently lower in Group D/E compared to Group P, Group E, and Group D, with no significant differences observed among the other groups [27].

In another RCT by Altıparmak et al. [28], the analgesic effect of duloxetine was evaluated in 64 patients. Patients were randomly assigned to 3 groups: (1) pregabalin 75 mg 1 hour before surgery, 12 hours after and 24 hours after, (2) duloxetine 60 mg 1 hour before and 24 hours after surgery, and (3) placebo. Postoperative pain was evaluated using VAS first minute, 30 minutes, 1, 12, 24, and 48 hours after surgery. The findings of this RCT showed similar pain scores in pregabalin and duloxetine groups, which were significantly lower than placebo group (first minute: D = 0 (0, 1), P = 0 (0, 0), C = 0 (0, 1), p = .049, 30 minutes: D = 2 (2, 3), P = 2 (1, 2), C = 3 (2, 3), p = .001, 1 hour: D = 3 (3, 4), P = 3 (3, 4), C = 4 (4, 5), p = .001, 24 hours: D = 3 (2, 4), P = 3 (2, 4), C = 4 (4, 4), p = .001, 48 hours: D = 2 (1, 3), P = 2 (1, 3), C = 3 (3, 4), p = .001) [28]. Moreover, Hyer et al. [29] randomly assigned patients to duloxetine + opioid and placebo + opioid groups. Treatment was started 2 weeks before surgery and continued until 3 months after surgery. Brief pain inventory was used for pain assessment 4 and 12 weeks postsurgery. The duloxetine group had significantly lower pain score postsurgery (p = .001) [29].

Bedin et al. [30] randomly assigned 57 patients to placebo and 60 mg duloxetine 1 hour before surgery and the following morning after surgery. Pain was assessed using verbal numeric scale 2, 6, 12, 24, 36, and 48 hours after surgery. The results showed no significant difference in pain scores between groups (p > .05) [30]. Moreover, Hiroki et al. [31] conducted an RCT and randomly assigned patients to single dose duloxetine 40 mg or diazepam (control) 4 mg 1 hour before surgery. VAS was used to evaluate pain 2, 3, 5, 10, 18, 21, 27, 42, 45, and 51 hours after surgery. The findings of this study indicated no significant difference in pain scores between duloxetine and control groups (p = .139) [31].

### Pilot and retrospective studies

Prabhala et al. [26] conducted a pilot trial to evaluate the effect of duloxetine 30 to 60 mg on pain in 108 patients. Pain was evaluated 12 months after surgery using MPQ, NRS and pain catastrophizing scale. The results showed that patients receiving duloxetine had better pain relief in the affective component of MPQ (0.18 ± 0.14, 0.54 ± 0.15, p = .046) [26]. No other measures showed significant differences [26].

Finally, in a retrospective observational study by Tsuji et al. [1] effects of duloxetine for postsurgical chronic neuropathic disorders was described. Patients were administered a daily dose range of 20–60 mg of duloxetine according to each patient's degree of adverse effects for more than 3 months. NRS was used to evaluate pain 3 months post-treatment initiation. Patients were categorized into 2 groups based on a 2-point or greater reduction in their NRS scores after duloxetine administration. Those with a ≥2 point decrease were considered as the effective group (E group), whereas others were classified as the not effective group (N group). A total of 19 patients (E group) had a positive response to duloxetine (rate of efficacy=79.2%). In particular, 100% of patients with postsurgical chronic pain were in the effective group (n = 9). The mean NRS pain scores significantly decreased in the E group (from 6.22 ± 1.92 at baseline to 2.78 ± 2.44 at 3 months). The mean NRS numbness score went from 6.34 ± 2.01 at baseline to 4.13 ± 2.44 after 3 months in all patients with numbness (n = 23). In the E group, NRS numbness scores decreased from 6.33 ± 1.68 to 3.56 ± 2.01, whereas in the N group, they changed from 6.40 ± 3.21 to 6.20 ± 2.95. Moreover, in the E group, both NRS pain and numbness scores significantly decreased (p < .01). Approximately 66.7% of patients with postsurgical chronic pain experienced marked improvement, whereas 33.3% had much-improved pain after duloxetine treatment [1].

### Postoperative opioid consumption

Total morphine consumption postsurgery was evaluated in 5 studies [1,^27^,[29], [30], [31]. Ongoing concomitant tramadol usage was assessed in Tsuji et al. [1] study which indicated no significant difference between group E and N (number of patients in E group: 4 vs. number of patients in N group: 1, p > .05). Furthermore, in Hiroki et al. (p = .930) and Hyer et al. (p = .307) studies, fentanyl use and opioid use was similar between groups [29,31]. However, the findings of Attia et al. and Bedin et al. indicated significant reduction in opioid consumption in patients receiving duloxetine (p = .001) [27,30].

### Adverse events

Attia and Mansour [27] reported higher incidence of nausea and vomiting in the placebo group in comparison with the D/E group. However, the difference between groups was not significant. Also, every patient who expressed these adverse events responded positively to intravenous ondansetron [27]. These findings were similar to Altiparmak et al. [28] study which reported higher incidence of nausea in the duloxetine group compared with the control group that was not statistically significant. Additionally, in Bedin et al. [30] study, patients in the duloxetine group reported headache, nausea, itching, and drowsiness which was not statistically different from the control group. Overall, duloxetine was well tolerated without serious adverse events.

### Quality assessment

The results of quality assessment are indicated in Fig. 1. Included RCTs had high quality in terms of randomization process, deviations from intended interventions, missing outcome data, measurement of the outcome, and selection of the reported result.

## Discussion

In this study, we conducted a systematic identification, review, and evaluation of published research investigating the analgesic impact of duloxetine in patients who have undergone spine surgery. Seven studies with a total of 487 patients were included. Duloxetine was generally well tolerated without serious adverse events or safety and tolerability concerns [27,^28^,30].

The results of the included studies offer valuable insights into the potential effectiveness of duloxetine for postoperative pain management. Notably, an RCT by Attia and Mansour [27] demonstrated that the combination of duloxetine and etoricoxib (D/E) significantly reduced pain scores across all time points, providing robust evidence for its analgesic effect. This finding suggests that combining these medications may be particularly advantageous in postoperative pain management. In contrast, Altıparmak et al. [28] indicated that duloxetine and pregabalin produced similar pain scores, both superior to the placebo group. Although this suggests that duloxetine and pregabalin may be interchangeable in certain contexts, further research is needed to determine specific patient populations where one might be favored over the other. The study conducted by Hyer et al. [29] underscored the potential benefits of initiating duloxetine treatment preoperatively, as it led to significantly lower pain scores post-surgery. This finding suggests that proactive pain management strategies involving duloxetine warrant consideration. However, the results from an RCT by Bedin et al. [30] did not demonstrate a significant difference in pain scores between the duloxetine and placebo groups. This outcome suggests that duloxetine's efficacy may not be consistent across all surgical pain scenarios, highlighting the need for a more nuanced understanding of its application. Similarly, Hiroki et al. [31] found no significant difference in pain scores between the duloxetine and control groups, indicating that duloxetine may not consistently provide pain relief in various postoperative contexts. These findings emphasize the importance of assessing duloxetine's efficacy on a case-by-case basis.

Prabhala et al. [26] conducted a pilot trial that provided a unique perspective, indicating that duloxetine may have a specific effect on the affective component of pain, as evidenced by improved pain relief in this aspect. Notably, this positive finding was limited to the affective component of pain in their study. Additionally, Tsuji et al. [1] performed a retrospective study describing the potential benefits of duloxetine for postsurgical chronic neuropathic disorders. Although this study reported positive outcomes, its retrospective nature requires cautious interpretation. There is also a potential bias due to dividing patients into successful and unsuccessful groups in this study. The collective evidence from these studies provides valuable insights into the potential of duloxetine as a postoperative pain management option. However, the variable results across studies and differences in methodology underscore the need for tailored approaches and further research to identify the specific patient populations and contexts where duloxetine may offer optimal pain relief. Critical analysis of study methodology reveals the importance of considering factors such as randomization procedures, blinding, and sample size when interpreting these findings.

The results of our review are consistent with previous meta-analyses and reviews that evaluated duloxetine's efficacy on osteoarthritis, chronic low back pain, and elective orthopedic surgeries [32,33]. As aforementioned, pain following surgery can result in increased mortality, morbidity, incidence of complications, as well as prolonged postoperative rehabilitation [3,4]. Therefore, it is essential to find the best strategies to control pain following spinal surgery. It should also be noted that studies on pain are challenging, since pain and its understanding are subjective, vary from individual to individual, and are impacted by a person's culture, experiences, age, gender, and ethnicity [34].

Some studies have only suggested duloxetine for treatment of chronic pain since its analgesic effect typically starts after 7 to 14 days of administration [35]. This can explain the insignificant results in Bedin et al. (2017) and Hiroki et al. (2022) studies [30,31]. Future studies should aim to optimize time schedules to maximize the analgesic effect and concurrently investigate the influence of patient-specific factors, such as age, gender, and comorbidities, on individual responses to duloxetine. This comprehensive approach can facilitate the personalized and more effective use of this medication while potentially identifying predictors of improved analgesic outcomes.

The current study offers a comprehensive exploration of duloxetine's effectiveness in managing postoperative pain after spinal surgery, but several limitations warrant consideration. First, the small number of included studies and limited sample sizes may impact the robustness of our conclusions. The diversity in surgical types, anesthesia methods, multimodal analgesia regimens, duloxetine doses, administration timings, and outcome measurement timelines introduces significant heterogeneity, making direct result comparisons challenging. The variability in duloxetine doses and treatment durations among the studies further complicates the analysis and may introduce bias. Additionally, inconsistent reporting of pain scale details in some studies limits precision. Publication bias and inherent limitations within individual studies add complexity to interpreting outcomes. There was a notable disparity in the composition of control and placebo groups in included studies. Specifically, the placebo group received varied treatments, including opioids in some cases and no opioids in others, which may introduce confounding variables that could affect the study's outcomes. The inclusion of diverse research methods and differences in treatment strategies and patient characteristics across studies also influence findings. Last, due to study heterogeneity and inadequate data, conducting a meta-analysis was not feasible, representing a substantial limitation. Although these limitations are acknowledged, their impact on the validity of our conclusions and the absence of quantitative data analysis due to heterogeneity should be considered when interpreting the results of this systematic review.

In conclusion, the findings suggest that duloxetine holds promise as an effective component and an alternative to opioids for postoperative pain management following spinal surgery. Although the results of the included studies collectively indicate a positive effect on pain control, it is crucial to recognize the potential limitations within individual studies and the variability in methodologies. To establish duloxetine's efficacy and safety conclusively, future RCTs should systematically compare it with other oral medications such as nonsteroidal anti-inflammatory drugs, assess its long-term safety profile, and determine the optimal dosage regimen. Furthermore, enhancing the robustness of future research through increased sample sizes and improved patient follow-up will contribute to more reliable and generalizable results, ultimately providing clinicians with a clearer understanding of duloxetine's role in optimizing postoperative pain relief for individuals undergoing spinal surgery.

## Declarations of competing interests

The authors declare that they have no known competing financial interests or personal relationships that could have appeared to influence the work reported in this paper.
